# Cortical Granule Exocytosis Is Mediated by Alpha-SNAP and N-Ethilmaleimide Sensitive Factor in Mouse Oocytes

**DOI:** 10.1371/journal.pone.0135679

**Published:** 2015-08-12

**Authors:** Matilde de Paola, Oscar Daniel Bello, Marcela Alejandra Michaut

**Affiliations:** 1 Instituto de Histología y Embriología, Consejo Nacional de Investigaciones Científicas y Tecnológicas, Universidad Nacional de Cuyo, Mendoza, Argentina; 2 Facultad de Ciencias Exactas y Naturales, Universidad Nacional de Cuyo, Mendoza, Argentina; Institute of Zoology, Chinese Academy of Sciences, CHINA

## Abstract

Cortical granule exocytosis (CGE), also known as cortical reaction, is a calcium- regulated secretion that represents a membrane fusion process during meiotic cell division of oocytes. The molecular mechanism of membrane fusion during CGE is still poorly understood and is thought to be mediated by the SNARE pathway; nevertheless, it is unkown if SNAP (acronym for soluble NSF attachment protein) and NSF (acronym for N-ethilmaleimide sensitive factor), two key proteins in the SNARE pathway, mediate CGE in any oocyte model. In this paper, we documented the gene expression of α-SNAP, γ-SNAP and NSF in mouse oocytes. Western blot analysis showed that the expression of these proteins maintains a similar level during oocyte maturation and early activation. Their localization was mainly observed at the cortical region of metaphase II oocytes, which is enriched in cortical granules. To evaluate the function of these proteins in CGE we set up a functional assay based on the quantification of cortical granules metaphase II oocytes activated parthenogenetically with strontium. Endogenous α-SNAP and NSF proteins were perturbed by microinjection of recombinant proteins or antibodies prior to CGE activation. The microinjection of wild type α-SNAP and the negative mutant of α-SNAP L294A in metaphase II oocytes inhibited CGE stimulated by strontium. NEM, an irreversibly inhibitor of NSF, and the microinjection of the negative mutant NSF D1EQ inhibited cortical reaction. The microinjection of anti-α-SNAP and anti-NSF antibodies was able to abolish CGE in activated metaphase II oocytes. The microinjection of anti-γ SNAP antibody had no effect on CGE. Our findings indicate, for the first time in any oocyte model, that α-SNAP, γ-SNAP, and NSF are expressed in mouse oocytes. We demonstrate that α-SNAP and NSF have an active role in CGE and propose a working model.

## Introduction

Mammalian fertilization is a process of fusion between a spermatozoon and an oocyte to create a zygote. To guarantee the success of fertilization and embryo development a definitive block to polyspermy is necessary since polyspermy is embryonic lethal. At least three postfertilization blocks to polyspermy have been described in mice. The first two occur rapidly and their molecular basis remains largely unknown, and the third, slow and definitive, correlates with the exocytosis of cortical granules in Metaphase II (MII) oocytes [1].

Cortical granules exocytosis in mouse oocytes, also known as cortical reaction, is a calcium regulated exocytosis. The cortical reaction differs from other exocytotic events in that cortical granules release occurs only once after oocyte´sfertilization and they are not renewed. The membrane fusion during this particular secretory process is thought to be mediated by SNAREs. However it is unknown if two essential proteins of membrane fusion, SNAP (acronym for soluble NSF attachment protein) and NSF (acronym for N-ethilmaleimide sensitive factor), are involved in the molecular mechanism of membrane fusion during cortical granule exoytosis (CGE).

The principle of action of the “SNARE hypothesis”, was formulated by Sollner and co-workers [2]. SNAREs (Soluble NSF-attachment protein receptors) are classified into vesicle (v)- and target-membrane (t)-SNAREs according to their localizations [2], or Arginine (R)- and Glutamine (Q)-SNAREs based on a key residue in the center of their SNARE domains [3]. There are two types of t-SNAREs: syntaxin-type and SNAP-25-type, and one v-SNARE: vesicle associated membrane proteins(VAMP)-type. SNARE proteins are the minimal machinery for membrane fusion and drive the fusion of biological membranes [4]. This process requires the formation of a protein complex that includes two t-SNAREs (on target membrane) and one v-SNARE (on vesicle membrane) known as the ternary trans-SNARE complex [5]. This assembly is thought to pull the fusing membranes closely together, driving bilayer fusion. After membrane fusion this tight SNARE complex-cis-SNARE complex- remains in the plasma membrane after exocytosis and needs to be disassembled and recycled to enable another round of fusion events. The disassembly of cis-SNARE complex is mediated by NSF, and the interaction between the SNARE complex and NSF requires SNAPs. Although overall sequence similarity between SNARE subtypes is limited, all SNARE complexes are disassembled by α-SNAP and NSF [6,7]. α-SNAP is recruited from the cytoplasm to the cis-SNARE complex in the membrane, and this SNAP/SNARE complex recruits NSF. α-SNAP regulates the ATPase activity of NSF and NSF, a chaperone-like ATPase, utilizes energy from ATP hydrolysis to dissociate cis-SNARE complex [7].

Although the importance of α-SNAP and NSF is unquestionable, the precise role of these key proteins in the membrane fusion during exocytosis is still a matter of debate. Given that membrane fusion can be visualized as a cycle, the role of α-SNAP and NSF can be seen as either the final event of one round of a cycle or the initial phase of the next. In effect two models have been proposed: the pre-fusion and the post-fusion model. The pre-fusion model postulates that α-SNAP and NSF disassemble cis-SNARE complex before fusion to allow the formation of trans-SNARE complex [8–11]. The post-fusion model postulates that SNAP/NSF is required to disassemble cis-SNARE after membrane fusion. This model is more accepted since in the comatose mutant in Drosophila, several rounds of vesicle fusion occur before the depletion of free SNARE causes neurotransmission to cease [12–15]

The signal-transducing pathway accountable for CGE is not yet completely understood and is thought to be mediated by SNAREs. In fact, in mouse oocytes, two proteins of this machinery have been characterized: SNAP-25 [16] and Syntaxin 4 [17]. In porcine oocytes, it has been documented that the SNAREs:Syntaxin 2, SNAP23, VAMP1, and VAMP2, are involved in cortical reaction [18]. Nevertheless, whether the chaperone NSF and its cofactor α-SNAP participate in CGE is still unknown. The aim of this work was to investigate the expression and localization of SNAPs isoforms and NSF in mouse oocytes and their participation in CGE. Here, we showed that that α-SNAP, γ-SNAP, and NSF are expressed in mouse oocytes, and that α-SNAP and NSF are required for cortical granule exocytosis.

## Materials and Methods

### Reagents

All chemicals, unless stated otherwise, were purchased from Sigma-Aldrich Chemical Inc. (St. Louis, USA).

### Animals, superovulation, and oocyte collection

This study was carried out in strict accordance with the recommendations in the Guide for the Care and Use of Laboratory Animals of the National Institutes of Health. The protocol was approved by the Institutional Animal Care and Use Committee of the School of Medicine of the National University of Cuyo(Protocol approval 252014). Female CF-1 mice (8–12 weeks) were kept in a room with 12 h/12 h light—dark cycles. GV oocytes were obtained from females primed by intraperitoneal (i.p.) injections with 10 IU of pregnant mare’s serum gonadotropin, PMSG (Syntex, Argentina), and 45–48 h later cumulus-oocyte complex were obtained by puncturing ovarian follicles. The collection medium was Earle’s balanced salt solution with 0.01% PVA, 0,001% Gentamycin, and 25 mM Hepes buffer, pH 7.3 (MEM/HEPES) supplemented with 2.5 μM Milrinone to inhibit oocyte maturation. Only GV oocytes about 80 μm in diameter and intact cumulus were used. After being pipetted repeatedly through a thin-bore pipette, cumulus cells were removed. MII oocytes were obtained from females primed with 10 IU (i.p.) of pregnant mare’s serum gonadotropin, PMSG (Syntex, Argentina) followed by 10 IU (i.p.) of human chorionic gonadotropin, hCG (Syntex, Argentina) 48 hr later. MII oocytes were collected from the oviductal ampullae by the scratching method between 13–17 h after hCG injection into MEM/HEPES, and denuded of cumulus cells by a brief exposure to 0,04% hyaluronidase. Both GV oocytes and MII oocytes were cultured until use in drops of M16 medium under mineral oil at 37°C in a humidified atmosphere of 5% CO2 in air. For a better understanding of the procedures described in Materials and Methods see the scheme of the experimental design (S1 Fig).

### RNA Isolation and Reverse Transcription-PCR

Mouse Brain tissue was homogenized and total RNA was isolated using Trizol reagent (Invitrogen) as advised by the manufacturer. Total RNA from 50 pooled GV oocytes was extracted using the RNAqueous micro kit (Ambion) according to the manufacturer’s instructions. Total RNA isolated from the oocytes or 2 μg RNA from brain were then reverse transcribed into complementary DNA. First-strand cDNA was extended by mouse Moloney Leukaemia Virus Reverse Transcriptase (Promega) during incubation for 1 h at 42°C with 1μg of Oligo dT (Biodynamics) in a final 25 μl reaction volume. Minus RT reaction was performed to check the absence of contaminating residual DNA. After completion of the reaction, first-strand cDNAs were analyzed by end-point PCR. Primer sequences were obtained from the PrimerBank database and primer selection was performed to avoid amplification of genomic DNA, selecting primers annealing at splice junction (exon-exon boundary) or intron spanning primers using CLC Sequence Viewer software (CLC Bio, Quiagen). The primer sequences and their corresponding PrimerBank ID used were as follows: α-SNAP forward primer 5´-AGGCTGCCCAACTACACCTA-3´, reverse primer 5´-ATCAGACAGTTAATGGCCTCTTG-3´ (13385392a2); β-SNAP forward primer 5´-GGAATGCGTTTTGTCAAGCTG-3´, reverse primer 5´- ATTGTGAACCTCCCCATGTCT-3´ (29789104a2); γ-SNAP forward primer 5´-GAGAAAGCCAGCATGATGTACC-3´, reverse primer 5´- CCCTCGTACCAGCAGTCTG-3´ (12845594a2); NSF forward primer 5´-AGGAGGCTTGGTGCTAACAG-3´, reverse primer 5´-TCTGGTCTATTGGTCATTCCGA-3´ (31543349a1). The expected size of the PCR products was 115, 172, 207 and 191 pb for α-SNAP, β-SNAP, γ-SNAP, and NSF, respectively. The PCR was conducted with cDNA of 16 oocyte equivalents and 10 ng of brain cDNA for SNAPs isoforms, or with cDNA of 20 oocyte equivalents and 100 ng of brain cDNA for NSF. Each sample in a run consisted of mentioned cDNA, 1 μM of each primer forward and reverse, 200 μM dNTPs (Promega); 5 μl Green GoTaq Reaction Buffer 5X (Promega) and 1 U of GoTaq DNA Polymerase (Promega) in a 25 μl reaction volume. For SNAPs isoforms, all genes were compared from the same stock to avoid inter-assay template variations. cDNAs were amplified singularly. Negative controls were performed omitting the reverse transcriptase (RT-PCR control) and the template (PCR control). cDNA from mouse brain tissue was used as a positive control. The reaction conditions for SNAPs isoforms amplification were template denaturation and polymerase activation at 95°C for 2 min, followed by 30 cycles of 95°C denaturation for 30 sec, 55°C annealing for 30 s and 72°C extension for 1 min, and a final extension at 72°C for 5 min. NSF was amplified using a program of template denaturation and polymerase activation at 94°C for 3 min, followed by 32 cycles of 1 min at 94°C, 45 sec at 55,3°C, and 1,5 min at 72°C with a final extention at 72°C for 10 min. The reactions were carried out using the Mastercycler Personal (Eppendorf) PCR thermal cycler. PCR products were subjected to 2% agarose gel electrophoresis, and the amplified products were visualized either using ImageQuant *LAS-4000* (Fujifilm) for samples prepared with SYBR safe DNA gel stain (Invitrogen) or using UV light following ethidium bromide staining.

### Recombinant proteins

Plasmids encoding wild type α-SNAP and mutant α-SNAP L294A in pET28a (Stratagene) were a kind gift from Dr. C. Tomes. Plasmid pGEX-4T-1 (Amersham) encoding γ-SNAP was a generous gift from Dr. M. Tagaya (Department of Molecular LifeSciences, Tokyo University of Pharmacy and Life Sciences). The pET28a (Stratagene) construct encoding NSF wild type was generously provided by Dr. D. Fasshauer (Max-Planck Institute for Biophysical Chemistry, Göttingen, Germany). Plasmid pQE-9 (Qiagen) encoding mutant NSF D1EQ was a kind gift from Dr S. Whiteheart (University of Kentucky, Lexington, KY). DNA encoding α-SNAP, α-SNAP L294A, γ-SNAP, NSF and NSF D1EQ were transformed into E.coli BL21 (Stratagene) and protein expression was induced with 0,75 (NSF) or 1 mM IPTG (wild type α-SNAP, α-SNAP L294A, γ-SNAP and NSF D1EQ) for 3 h at 37°C. Purification of His6-tagged recombinant proteins was carried out under native conditions according to Qiagen’s instructions with the exception that the purification buffers contained 20 mM TrisHCl, pH 7.4, instead of 50 mM phosphate, pH 8. In all buffers NaCl concentration was 200 mM. Lysis buffer contained 10 mM imidazole, washing buffer contained 20 mM imidazole and elution buffer contained 250 mMimidazole. Recombinant wild type NSF and mutant NSF D1EQ were prepared and purified essentially as previously described [19,20]. Glutathione S-transferase (GST) fusion protein γ-SNAP was generated and purified according to protocols described in the GST Gene Fusion System instructions (Pharmacia). GST tag was removed from free eluted γ-SNAP-GST fusion protein using 10 U Thrombin (MP Biomedicals) per mg of purified protein, overnight at 22°C under shaking. Protein determination was assesed by Bradford method (Biorad) in 96-well microplates. Bovine serum albumin was used as a standard and the results were quantified on a Multiskan FC (Thermo Scientific) microplate reader. For microinjection, proteins were desalted by Gel filtration using Sephadex G-25 (MP Biomedicals). In the case of NSF D1EQ, efficient desalting was achieved using a Microcon YM-30 centrifugal filter device (Millipore) according to the manufacturer’s instructions.

### Immunoblotting

GV oocytes, MII oocytes and strontium activated MII oocytes were placed in Laemmli sample buffer containing 200 mM DTT and heated for 5 min at 100°C prior to immunoblotting. As positive controls total proteins from mouse brain extract and purified recombinant proteins were prepared. Proteins were separated in a 4% stacking and 12 or 15% running SDS-PAGE gel (see figure legend), and electrotransferred onto a PVDF membrane sheet Immobilon-P (Millipore) using wet transfer. Membranes were blocked in 2% BSA or 2% ECL Advance Blocking Agent (GE Healthcare) in TBS containing 0.1% Tween 20, (TTBS) for 1 h at RT. After blocking, blots were individually incubated overnight at 4°C with primary antibodies diluted in blocking solution at the indicated final concentrations: monoclonal anti-α-/β-SNAP antibody (0,2 ng/μl, Synaptic Systems, clone 77.2), rabbit polyclonal isotype-specific anti-γ-SNAP antibody (1 ng/μl, Synaptic Systems), and rabbit polyclonal anti-NSF antibody (1:1000 dilution, Synaptic Systems). The presence of β-Tubulin and β-Actin were detected with mouse monoclonal antibodies anti-β-Tubulin (1:2000 dilution, Sigma-Aldrich, clone TUB 2.1) and anti-β-Actin (1 ng/μl), respectively. After washing 3 times in TTBS, the membranes were incubated for 1 h at RT with goat anti-mouse IgG-HRP antibody (80pg/μl, Jackson ImmunoResearch Laboratories) or goat anti-rabbit IgG-HRP antibody (1:10000, Cell Signaling Technology). After washing, the signal was detected using ECL Advance Western Blotting System (GE Healthcare) and visualized usingImageQuant *LAS-4000* (Fujifilm).

Densitometry analysis was conducted using the public sector image processing program Image J (version 1.42l; NIH, MD) and values were normalized to the loading control. Specificity of the primary antibodies was confirmed by preabsorbing the antibodies with their control peptide antigens in the case of anti-γ-SNAP and anti-NSF antibodies (Synaptic Systems) or full lenght α-SNAP recombinant protein for anti-α/β-SNAP antibody. For neutralization, the antibodies were combined with a fivefold excess of blocking agents in 1 ml of TBS for 2 h with agitation at room temperature (RT), and then diluted into appropriate final working voulume to proceed with the Western blot procedure as usual.

### Immunofluorescence

Oocytes or 1 cell embryos were briefly exposed to acidic Tyrode’s solution pH 2.2, to remove the zona pellucida and fixed in 3,7% paraformaldehyde in Dulbecco’s PBS (DPBS) for 1h at RT.

Fixed cells were washed in blocking solution (BS) containing 3 mg/ml BSA, 100 mM glycine and 0,01% Tween 20 in DPBS before permeabilization with 0.1% Triton-X in DPBS for 15 min at RT. Following permeabilization, oocytes were washed 3 times in BS and incubated with primary antibodies overnight at 4°C. The same primary antibodies describe above were used for immunocytochemistry at the indicated final concentrations: anti-α-/β-SNAP (50 ng/μl) anti-γ-SNAP (100 ng/μl), and anti-NSF (1:20). After washing, cells were incubated with the secondary antibody at RT for 1 h. The secondary antibodies used were DyLight 488 donkey anti-mouse (3ng/μl, Jackson InmunoReasearch) and DyLight 488 donkey anti-rabbit (3ng/μl, Jackson InmunoReasearch). After washing, cells were incubated in 25 μg/ml rhodamine labeled Lens Culinaris Agglutinin (LCA) in BS for 30 min for CG labeling, and mounted in Vectashield Mounting Medium (Vector Laboratories, Burlingame, CA) containing 1.5 μg/ml Hoechst 33342 (Molecular Probes, Invitrogen) for DNA detection on a slide under minimal compression, sealed, and stored at 4°C until visualization. Nonspecific staining was determined by incubation without primary antibody. Each experiment was performed at least 3 times for each condition. Images were obtained at the equatorial region of the cells using a FV1000 Confocal Microscope (Olympus), with a PLAPON 60x/NA1.42 oil-immersion objective lens, at 512 x 512 pixel resolution. For each experimental series, images were captured using the same microscope settings. ImageJ software (version 1.42l; NIH, MD) was used for the analysis of the images.

### In vitro fertilization

For in vitro fertilization, sperm and MII oocyte culture, as well as co-incubation were carried out into human tubal fluid (HTF) containing 5 mg/ml BSA covered with mineral oil, at 37°C in a humidified atmosphere of 5% CO_2_ in air. Spermatozoa were obtained from adult CF-1 males (3–6 months old) with proven fertility by excising the cauda epididymis. After incubation for 15 min, the sperm concentration was adjusted to 5 x 10^6^ sperm/ml and incubated for 2 h in HTF for capacitation. For co-incubation, the sperm suspension was diluted to obtain 100 μl insemination drops containing 1–5×10^4^ spz/ml. MII oocytes were collected from superovulated females as described above. 10–20 MII oocytes were incubated in each drop. After 6–7 hs of insemination, oocytes were washed using a thin-bore pipette to remove loosely attached sperm before fixation.

### Artificial activation of Metaphase II oocytes

After collection, MII oocytes were parthenogenetically activated by either calcium ionophore A23187 or SrCl_2_. For ionophore activation, the oocytes were incubated in 20 μM A23187 for 2 minutes in M16 medium, thoroughly washed in calcium/magnesium-free CZB medium and incubated in the same medium for 30 minutes. For SrCl_2_ treatment, the oocytes were thoroughly washed in calcium/magnesium-free CZB, and activated by freshly prepared SrCl_2_ (10–30 mM) in the same medium, during 1 h. All incubations were performed at 37°C in a humidified atmosphere of CO_2_ (5%) in air. After activation, oocytes for CGE assays were inmediately processed for CG staining and in the case of inmuno staining, some oocytes were processed (1h SrCl_2_) and some incubated for additional 6 hs (7hs SrCl_2_) in regular M16 medium.

### NEM treatment

MII oocytes were exposed to either 10 or 50 μM NEM in M16 Medium for 5 minutes at 37°C in a humidified atmosphere with 5% CO_2_, thoroughly washed and then used in CG exocytosis experiments. The number of oocytes used for each experiment is indicated in the figure legends.

### Oocyte Microinjection

MII oocytes were microinjected with 10 μM purified recombinant proteins (wild type α-SNAP, mutant α-SNAP L294A, wild type NSF and mutant NSF D1EQ) diluted in PBS if necessary. Anti-α-/β-SNAP, anti-γ-SNAP, and anti-NSF antibodies (Synaptic Systems) were microinjected at the maximum possible concentration. Anti-α-/β-SNAP, anti-γ-SNAP, mouse IgG isotype control (Novus Biologicals), and rabbit IgG isotype control (Novus Biologicals) were microinjected at 1 μg/μl. All antibodies and isotype controls were prepared in PBS. Microinjection pipettes were made by pulling borosilicate-glass capillary tubing in a mechanical puller (model P-97; Sutter Instrument Co., Novato, CA). Microinjections were performed using micromanipulators (Narishige) coupled to a Olympus IX-51 inverted microscope (Olympus). All micromanipulations were carried out in 5 μl drops of MEM/HEPES. For microinjection, needles were filled with injection solutions at the indicated concentrations, and about 7–10 pl was injected into the cytoplasm of MII oocytes by pneumatic pressure using a Pico-Injector (model PLI-100, Harvard Apparatus, Holliston, MA). Injected oocytes were used in CG exocytosis experiments after at least 1 h incubation in M16 medium, in a humidified atmosphere with 5% CO_2_ at 37°C. The number of oocytes used for each experiment is indicated in the figure legends.

### Cortical granule staining and quantification

Cortical granule staining and quantification were performed according to Ducibella et al [21]. The zona pellucida was removed by brief incubation in acid Tyrodes, pH 2.2, washed in MEM/HEPES and fixed in 3.7% paraformaldehyde in Dulbecco’s PBS (DPBS) for 1h at RT. After fixation, cells were washed in BS and permeabilized with 0.1% Triton X-100 in DPBS for 15 min. After 3 washes in BS, cellswere incubated in 25μg/ml FITC-LCA in BS for 30 min, and mounted in Vectashield MountingMedium (Vector Laboratories, Burlingame, CA) containing 1.5 μg/ml Hoechst 33342 (Molecular Probes, Invitrogen) or 1 μg/mlpropidium iodide (Molecular Probes, Invitrogen) for DNA labeling on a slide, sealed, and stored at 4°C until visualization. The images on flat optical fields of cortex resulting from partial compression of cells by the coverslip were acquiredwith a confocal laser-scanning microscope (FV1000, Olympus) using a PLAPON 60x/NA1.42 oil-immersion objective lens, at 512 x 512 pixel resolution. The confocal acquisition parameters remained constant for all captured images within the same experiment. The cortical granules (CG) density per 100 μm^2^ (CG/100 μm^2^) for each cell was determined as the mean of the counts from at least four non overlapping equal areas of cortex containing cortical granules, according to Ducibella et al. (1988), by computer-assisted image quantification using Image J (version 1.42l; NIH, MD). For each group, relative CG density/100 μm^2^ was calculated by comparing the mean density of CGs of the treated group with the mean density of CGs of the untreated control group, according to the following equation: [density of CGs in treated group/density of CGs in untreated group] x100, thus setting density of CGs in untreated group (control condition) as 100%.

### Data Analysis

Experiments were repeated at least three times. The number of oocytes used for each experiment is indicated in the figure legends. Data analysis was performed using KyPlot software. Statistical significance was determined by Student’s t test, or One-Way Analysis of Variance (ANOVA) followed by Tukey’s test for multiple comparison. Data are expressed as mean ± SEM and p < 0.05 is considered statistically significant. Statistical significances in frequency histograms were performed using the Kolmogorov-Smirnov test for two sets of data and p < 0.05 is considered statistically significant.

## Results and Discussion

### Gene expression of SNAP isoforms and NSF in mouse oocytes

**Fig 1:**
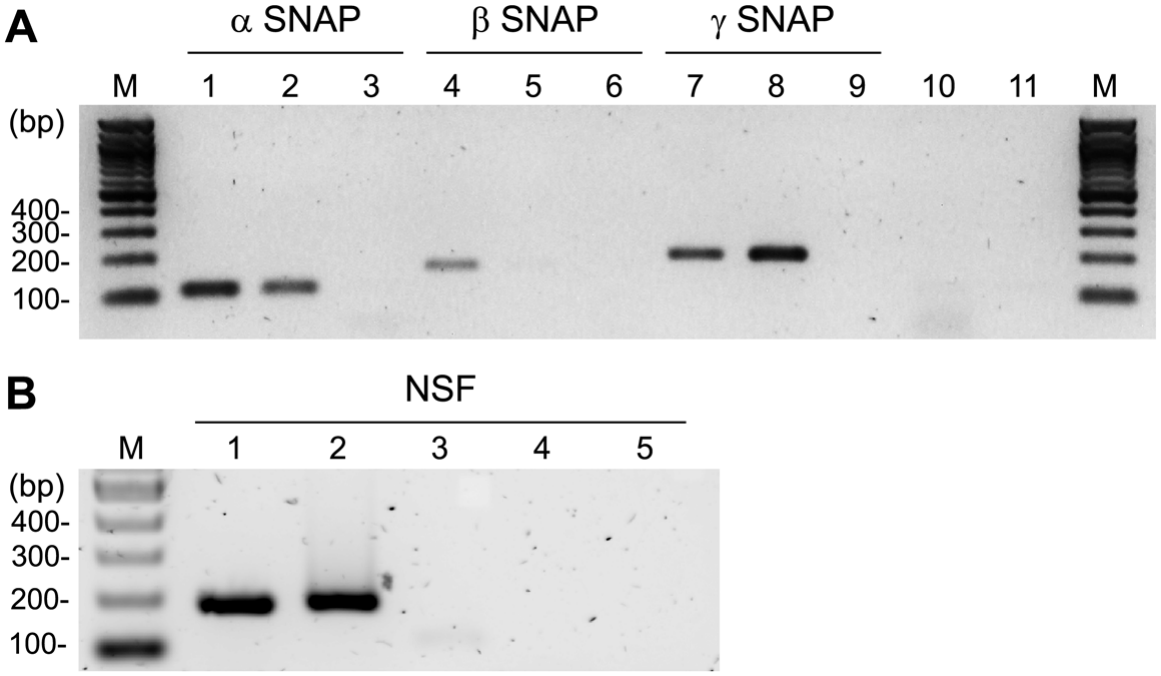
Gene Expression of SNAP isoforms and NSF in mouse oocytes. **A**. Detection of SNAPs mRNA. Lanes 1–3 and 10–11 were amplified using α-SNAP primers, lanes 4–6 using β-SNAP primers, and lanes 7–9 using γ-SNAP primers under the same experimental procedure. Agarose gel was stained with ethidium bromide. Lanes: M, molecular weight marker; 1,4, and 7,mouse brain; 2, 5, and 8, GV oocytes; 3, 6, and 9, PCR negative controls without cDNA; 10 and 11, RT-PCR negative controls without reverse transcriptase (RT) for brain and oocytes samples, respectively. **B**. Detection of NSF mRNA. All lanes were amplified using NSF primers and agarose gel was stained with SYBR safe. Lanes: M, molecular weight marker; 1,mouse brain; 2, mouse oocytes; 3, PCR negative control without cDNA; 4 and 5, RT-PCR negative control without RT for brain and oocytes samples, respectively. Shown are images representative of 3 independent experiments.

Three isoforms of SNAP: α-, β- and γ-SNAP [22], and one isoform of NSF [23] have been described. To determine which of these proteins is expressed in mouse oocytes, the presence of mRNA for the three SNAP isoforms andNSF was assessed by RT-PCR. We reverse transcribed cDNA samples from mRNA isolated from germinal vesicle (GV)—intact oocytes and amplified them using the respective specific primers. Amplified products were observed in the α-SNAP (Fig 1A, lane 2), γ-SNAP (Fig 1A, lane 8) and NSF (Fig 1B, lane 2) lanes, showing that α-SNAP, γ-SNAP, and NSF mRNA but not that of β-SNAP (Fig 1A, lane 5) were expressed in GV-intact oocytes. Since the expression of SNAPs and NSF has already been studied in neuronal exocytosis, brain was used as positive control. Amplification products from mouse brain cDNA were observed in all lines of α-SNAP, β-SNAP, γ-SNAP, and NSF (Fig 1A: lanes 1, 4, and 7; Fig 1B: lane 1). No amplified DNA fragments were observed when PCR was performed without cDNA sample (Fig 1A, lanes 3, 6, and 9; Fig 1B, lane 3) or without reverse transcriptase (Fig 1A, lanes 10 and 11; Fig 1B, lane 4 and 5). These findings are in concordance with the fact that β-SNAP is specific for neuronal tissue, and α-SNAP and γ-SNAP are ubiquitously expressed [20]. Our results show that mRNA for α-SNAP, γ-SNAP and NSF are expressed in mouse oocytes. Hence, the expression and localization of these proteins were tested.

### Expression of α-SNAP, γ-SNAP and NSF during oocyte maturation and early activation

We determined the expression of α-SNAP, γ-SNAP and NSF proteins by Western blott during oocyte maturation and early activation. To analyze oocyte maturation we collected the following stages: GV-intact oocytes, Metaphase II (MII) oocytes, and strontium-activated MII oocytes. Proteins from different stages were resolved by SDS-PAGE, transferred to PVDF membranes and probed with the antibody of interest: anti-α-SNAP, anti-γ- SNAP, or NSF antibody.

**Fig 2:**
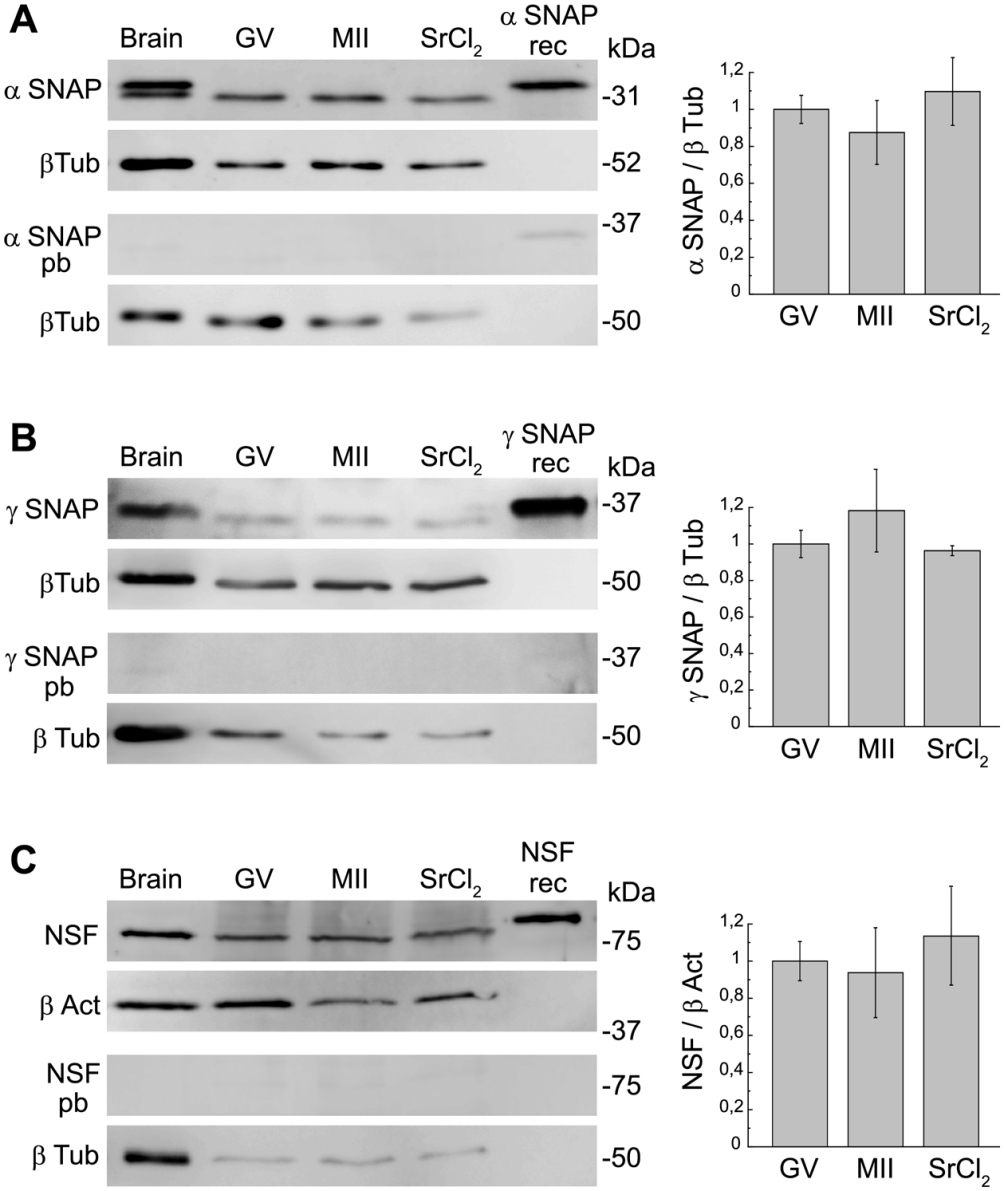
Detection of α-SNAP, γ-SNAP and NSF by Western blot. **A**. *Left*, *Upper panels*: Inmunoblot of α-SNAP: Protein extracts from equal numbers (150) of GV-intact oocytes (GV), MII oocytes (MII) and parthenogenetic activated MII oocytes with 10mM strontium chloride (SrCl_2_) were separated on a 12% SDS-PAGE gel. Positive controls: mouse brain (Brain, 1.25 μg) and recombinant His6-α-SNAP (α SNAP rec, 5 ng). Immunoblot of β- tubulin (β Tub) was performed as a control of protein loading. *Lower panels*: Immunoblot using anti-α-SNAP antibody preabsorbed with full lenght α-SNAP recombinant protein (α SNAP pb). *Right*, densitometry analysis of Western blots for α-SNAP (mean ± SEM, n = 4) showing α-SNAP protein expression level (α SNAP/β Tub ratio) relative to GV expression, set as 1. **B**. *Left*, *Upper panels*: Inmunoblot of γ-SNAP: Protein extracts from equal numbers (300) of GV-intact oocytes (GV), MII oocytes (MII) and parthenogenetic activated MII oocytes with 10mM strontium chloride (SrCl_2_) were separated on a 12% SDS-PAGE gel. Positive controls: mouse brain (Brain, 6 μg) and recombinant thrombine cleaved γ-SNAP-GST (γ SNAP rec, 0.2 μg). Immunoblot of β- tubulin (β Tub) was performed as a control of protein loading. *Lower panels*: Immunoblot using anti-γ-SNAP antibody preabsorbed with γ-SNAP control peptide (γ SNAP pb). *Right*, densitometry analysis of Western blots for γ-SNAP (mean ± SEM, n = 3) showing γ-SNAP protein expression level (γ SNAP/β Tub ratio) relative to GV expression, set as 1. **C**. *Left*, *Upper panels*: Inmunoblot of NSF: Protein extracts from equal numbers (200) of GV-intact oocytes (GV), MII oocytes (MII) and parthenogenetic activated MII oocytes with 10 mM strontium chloride (SrCl_2_) were separated on a 15% SDS-PAGE gel. Positive controls: mouse brain (Brain, 3,5 μg) and recombinant His6-NSF (NSF rec, 75 ng). Immunoblot of β- actin (β Act) was performed as a control of protein loading. *Lower panels*: Immunoblot using anti-NSF antibody preabsorbed with NSF control peptide (NSF pb). *Right*, densitometry analysis of Western blots for NSF (mean ± SEM, n = 3) showing NSF protein expression level (NSF/ β Act ratio) relative to GV expression, set as 1. In all panels MW protein standards (x10^3^) are indicated on the right and primary antibodies, on the left.

For α-SNAP detection, a monoclonal antibody raised against recombinant full length α-SNAP was used. The immunoblot analysis of GV-intact oocytes, MII oocytes and parthenogenetic activated oocytes demonstrated the presence of a single protein band, that comigrated with mouse brain and recombinant α-SNAP used as positive controls (Fig 2A, upper panel). This result indicated that α-SNAP is present in mouse oocytes. The antibody recognized a unique band for all conditions, except for mouse brain, which showed two bands because it expresses both α- and β-SNAP isoforms. Preincubation with an excess of recombinant α-SNAP protein abolished the signal, indicating that antibody was specific for α-SNAP (Fig 2A, lower panel). Densitometry analysis of α-SNAP expression for all cell stages showed no significant differences (Fig 2A, right panel).

For γ-SNAP detection, a polyclonal antibody raised against a γ-SNAP peptide (amino acid residues 2–18) was used. Western blot analysis showed that a single band of the expected molecular weight was present in all protein extractscorresponding to mouse brain, recombinant γ-SNAP and different stages of mouse oocyte (Fig 2B, upper panel). In this case, antibody specificity was tested by preincubation of γ-SNAP antibody with an excess of control peptide corresponding to amino acid residues 2–18. As shown in Fig 2B (lower panel), γ-SNAP signal was abolished after antibody preabsorbing, demonstrating that γ-SNAP antibody was specific for γ-SNAP. Quantification of γ-SNAP expression level through oocyte maturation and early activation showed no significant differences (Fig 2B, right panel).

Similar assays were performed for NSF detection. For immunoblotting analysis a polyclonal antibody raised against an NSF peptide (amino acid residues 733–744) was used. As shown in Fig 2C (upper panel), a single band of the expected molecular weight was observed in all analyzed samples: mouse brain (positive control), GV-intact oocytes, MII oocytes, strontium- activated MII oocytes, and recombinant NSF (positive control). Again, preincubation of NSF antibody with an excess of control peptide eliminated the signal, indicating that the observed band was specific for NSF (Fig 2C, lower panel) and densitometry analysis of NSF expression between different cell stages showed no significant differences (Fig 2C, right panel).

Altoghether, these results showed that α-SNAP, γ-SNAP and NSF proteins are expressed in mouse oocytes and their expression level remains constant during oocyte maturation and early activation.

### Localization of α-SNAP, γ-SNAP and NSF during oocyte maturation and activation

**Fig 3:**
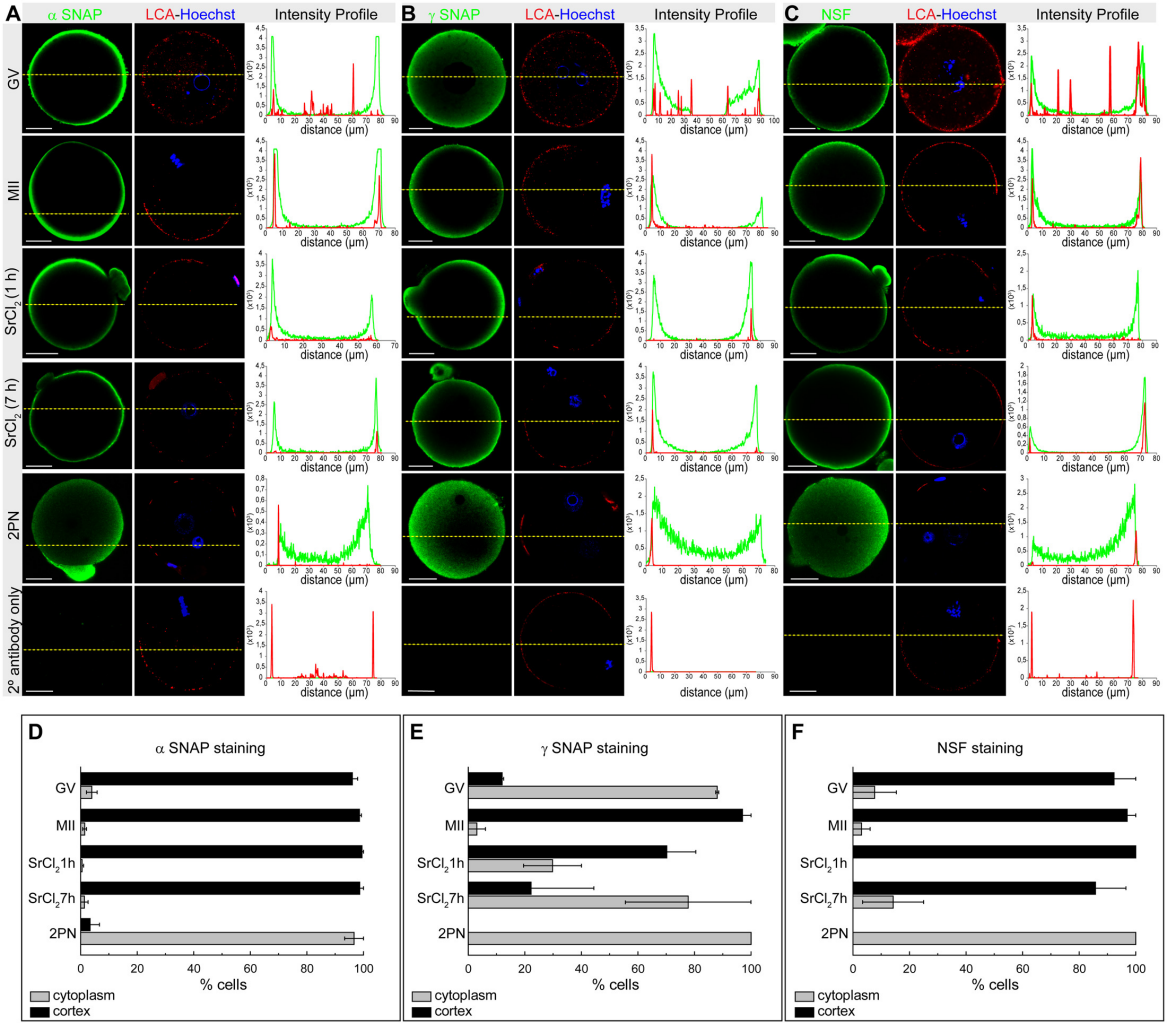
α-SNAP, γ-SNAP, and NSF localization during meiotic maturation and MII oocytes activation. α-SNAP (left panel), γ-SNAP (middle panel) and NSF (right panel) were immunodetected at different stages: GV-intact oocytes (GV), MII oocytes(MII), parthenogenetic activated MII oocytes with 10mM strontium chloride during 1 or 7 h post activation (SrCl_2_ (1h) and (7h), respectively), and two pronucleus (2PN) embryos after in vitro fertilization. Green, positive staining for primary α-/β-SNAP, γ-SNAP or NSF antibody detected by secondary antibodies conjugated to DyLight 488; red, cortical granules stained with LCA-Rhodamine; blue, DNA labeled with Hoechst 3342.**A-C**, panels show the fluorescence intensity profiles for α-SNAP, γ-SNAP and NSF (green) and cortical granules stained with LCA-Rhodamine (red). Fluorescence intensities were measured along dashed lines traced in each panel. The intensities of α-SNAP, γ-SNAP and NSF are indicated by green lines, and the intensity of cortical granules is indicated by red lines. Scale bar: 20 μm. **D**-**F**, cortical and cytoplasmic patterns of protein distribution. Cortical region was defined as the region of 10 μm thickness from the oocyte plasma membrane towards the oocyte centre. Those cells in which fluorescence decay at 10 μm were considered to present a cortical staining, and those cells which present high fluorescence at 10um towards the center and beyond were considered to present cytoplasmic staining. Percentage analysis was assessed. Number of analyzed cells for α-SNAP, γ-SNAP and NSF were, respectively: GV = 85,51,32; MII = 138,37,35; MII SrCl_2_ 1h = 101, 36, 30; MII SrCl_2_ 7h = 35, 27, 33; IVF = 13, 6, 9.

Next, we analyzed the localization of α-SNAP, γ-SNAP and NSF during oocyte maturation and activation. For immunolocalization studies we included two more stages of embryo development. Besides GV-intact oocytes, MII oocytes and strontium-activated MII oocytes during 1h, we also analyzed activated MII oocytes after 7h of strontium treatment and two pronuclei (2PN) embryos after in vitro fertilization. As shown in Fig 3, both α-SNAP and NSF staining were mainly concentrated in the cortex region, while γ-SNAP was localized in both cortical and cytoplasmic region (see also S2 Fig). To better analyze the distribution of proteins, an analysis of fluorescence intensity profiles was performed (Fig 3). Two staining patterns were defined: the cortical and the cytoplasmic pattern. Those cells in which fluorescence decay at 10 μm were considered to present a cortical staining and, those cells which showed fluorescence at 10um and beyond (towards the oocyte centre) were considered to present cytoplasmic staining. α-SNAP and NSF showed a cortical localization in more than 90% of GV-intact oocytes, MII oocytes, and strontium-activated MII oocytes (Fig 3D and 3F). When γ-SNAP localization was analyzed, it presented both cortical and cytoplasmic distribution, showing an important cytoplasmic localization in GV-intact oocytes (close to 90%, Fig 3E) and 2PN embryos (close to 100%, Fig 3E). The cytoplasmic localization of γ-SNAP in GV-intact oocytes and 2PN embryos is very interesting since these stages are the only ones that have nucleus-germinal vesicle in GV-intact oocytes and female and male pronucleus in 2PN embryos- during the progression of oocyte meiosis. Whether the cytoplasmic localization of γ-SNAP is relevant for its function remains to be explored. In addition, α-SNAP and NSF proteins also showed a significant cytoplasmic localization in 2PN embryos (over 95%, Fig 3D and 3F). As far as we know, the function of α-SNAP and NSF during meiotic division has not been studied.

Altogether, these results showed that α-SNAP, γ-SNAP, and NSF are localized in the cortical region of MII oocytes, which is enriched with cortical granules. This localization prompted us to investigate the involvement of these proteins in CGE.

### Only α-SNAP and NSF have an active role in CGE

The role of α-SNAP in membrane fusion during exocytosis is well established. This role has been defined mainly as an adaptor that allows NSF binding to SNAREs [24]. However, the function of the ubiquitously expressed γ-SNAP remains unclear. In permeabilized adrenal chromaffin cells, it has been reported that the addition of α- or γ-SNAP is able to stimulate calcium-dependent exocytosis [25]. In vitro studies showed that γ-SNAP, which only shares a 20% amino acid residues with α-/β-SNAP, can bind NSF independently of SNAREs, and this interaction is essential for γ-SNAP to be incorporated into the SNARE core complex [26]. In other words, unlike α-/β-SNAP, γ-SNAP does not bind SNAREs in a direct manner, γ-SNAP binds SNAREs via NSF [26].

**Fig 4:**
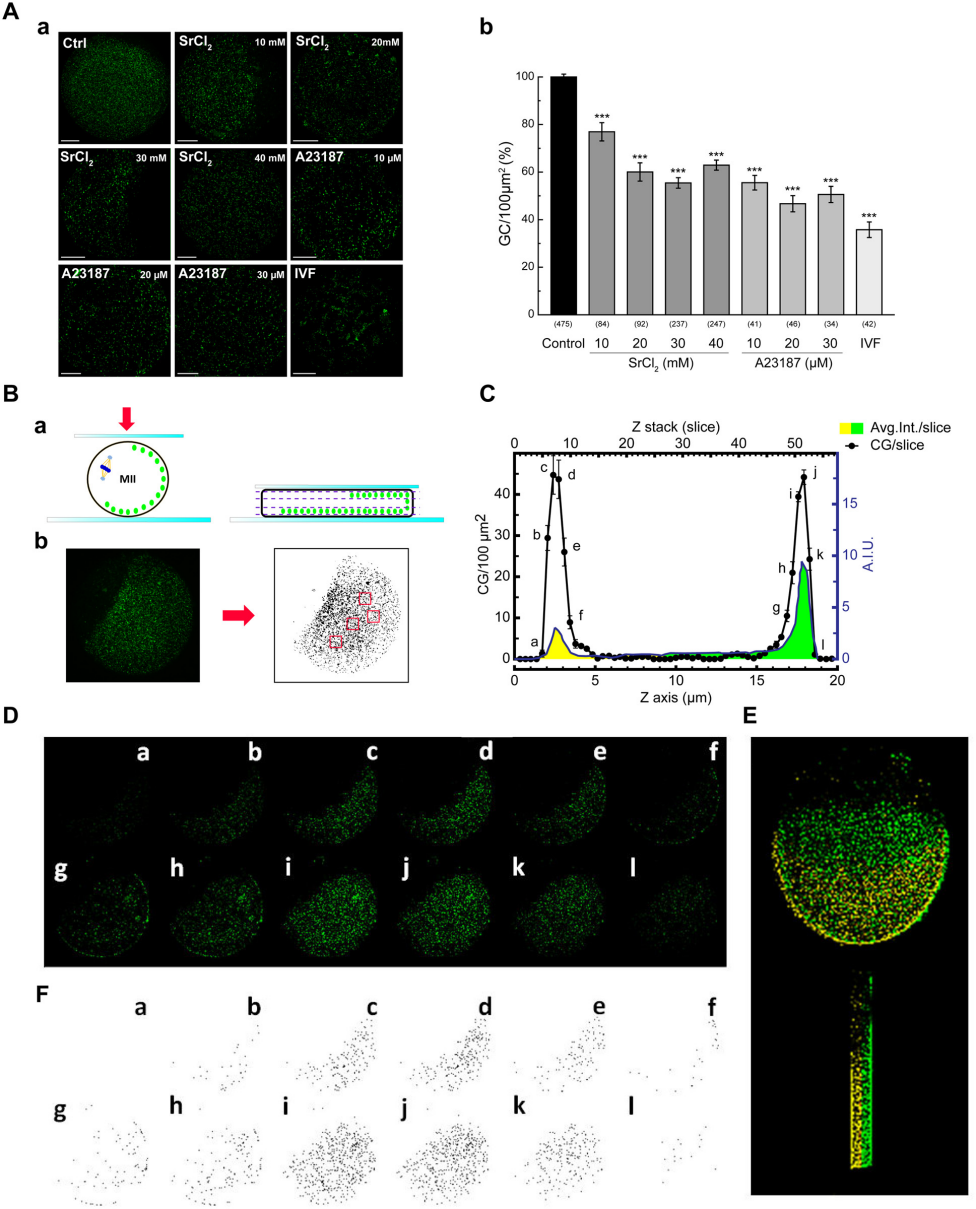
Functional assay of cortical granule exocytosis. **A**. Cortical granule exocytosis (CGE) was triggered bySrCl_2_ (10-40mM), A23187 (10–30 μM) or in vitro fertilization (IVF, cells fixed after 6 h co-incubation), and cortical granules (CG) were quantified. **a**, representative confocal images of oocytes subjected to different treatments, fixed and stained with FITC-LCA to label cortical granules. Scale bar: 20 μm. **b**, histogram shows CG density (% CG density/100 μm^2^) relative to untreated group (control) set as 100%. Data are shown as mean ± SEM from at least 3 independent experiments; numbers in parentheses below bars represents total number of activated MII oocytes (SrCl_2_, A23187) or embryos (IVF). ***, p ≤ 0.001 (Tukey’s test). **B**. **a**, schematic diagram depicting mounting of an MII oocyte partially compressed under a coverslip in order to provide a large flat field of cortical granules (purple dashed lines). **b**, confocal images on flat optical field, showing the biggest area containing cortical granules. Once image threshold was adjusted, this remained constant for all images within the same experiment. CG were computer-assisted counted from at least 4 non overlapping equal areas of 100 μm^2^ (red squares), and CG density/100 μm^2^ was determined as the mean of the counts. **C**. Cortical granules density is similar regardless the area size containing cortical granules and total fluorescence intensity. To show this, 58 serial pictures were taken with a spacingof 0.39μmin thezaxis (Z axis). CG density and fluorescence intensity measured as arbitrary intensity units (A.I.U) were plotted for each slice (Avg. Int./slice). **D**. Representative images for each slice (a-l) showed in **C**. **F**. Threshold of the images (a-l) shown in **D**, used for quantification of cortical granules. **E**. Tridimensional reconstruction of the MII oocyte from **D** panel showing cortical granules distribution. Top image, front view; bottom image, side view. Yellow and green colors represent images from slide and coverslip, respectively.

According to the well known function of SNAPs and NSF in membrane fusion during exocytosis ([11,24] and our localization findings, we hypothesized that α-SNAP, γ-SNAP, and NSF participate in CGE. To test this hypothesis, we attempted to perturb endogenous proteins during CGE using a functional assay. First, we set up cortical granules quantification using a methodpreviously described by Ducibella and collaborators [21]. We measured and compared cortical granules exocytosis triggered by physiological and parthenogenetic activators. For physiological activation, we performed in vitro fertilization using mouse sperm and for parthenogenetic activation, we assayeddifferent concentrations of A23187, a calcium ionophore, and strontium chloride (see Fig 4A). After each treatment, the zona pellucida was removed and cortical granules were stained with Lens Culinaris Agglutinin (LCA)-FITC to quantify cortical granule density. As shown in Fig 4, after in vitro fertilization, the denstity of cortical granules decreased 64% compared to control cells. CGE triggered by parthenogenetic activators, A23187 and strontium chloride, also decreased the density of cortical granulesand was concentration dependent. A23187 (30 μM) and strontium chloride (30 mM) diminished 48,4% and 42%, respectively, the cortical granules density compared to not activated MII oocytes.

**Fig 5:**
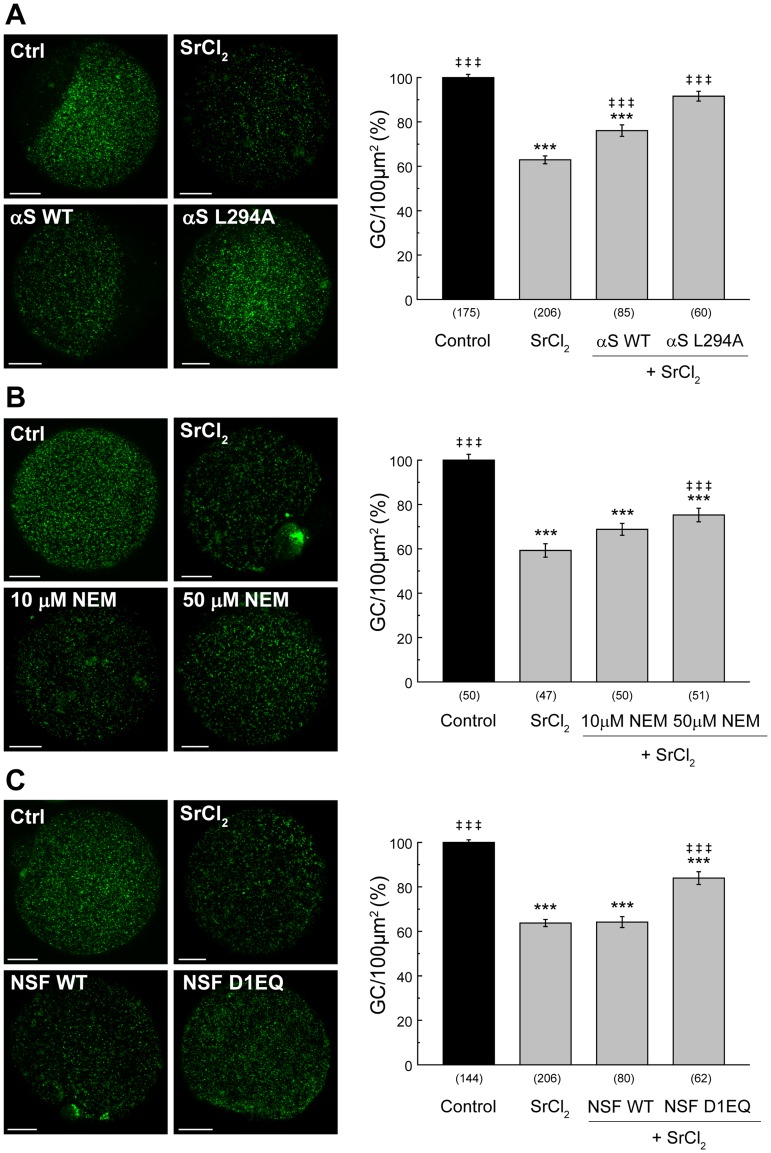
Effect of recombinant wild type α-SNAP, mutant α-SNAP L294A, NEM, wild type NSFand mutant NSF D1EQ and on CGE. **A**. MII oocytes were microinjected with wild type α-SNAP (αS WT) or mutant α-SNAP L294A (αS L294A) prior to CGE activation with 30 mM strontium chloride (SrCl_2_). *Left*, representative images of MII oocytes stained with FITC-LCA to label cortical granules. *Right*, histogram showing % CG density/100 μm^2^ relative to untreated group (Control) set as 100%. Data are shown as mean ± SEM from at least 5 independent experiments; numbers in parentheses represent total number of MII oocytes. ***, values compared to control, p ≤ 0.001;‡ ‡ ‡, values compared to SrCl_2_, p ≤ 0.001. Scale bar: 20 μm. **B**. MII oocytes were incubated in presence of different NEM concentrations prior to CGE activated by strontium chloride (SrCl_2_). Histogram shows % CG density/100 μm^2^ relative to untreated group (Control) set as 100%. Data are shown as mean ± SEM from at least 5 independent experiments.**C**. MII oocytes were microinjected with wild type NSF (NSF WT) or mutant NSF D1EQ (NSF D1EQ) prior to CGE activation with 30 mM strontium chloride (SrCl_2_). *Left*, representative images of MII oocytes stained with FITC-LCA to label cortical granules. *Right*, histogram showing % CG density/100 μm^2^ relative to untreated group (Control) set as 100%. Data are shown as mean ± SEM from at least 5 independent experiments; numbers in parentheses below bars represent total number of oocytes. ***, values compared to control, p ≤ 0.001;‡ ‡ ‡, values compared to SrCl_2_, p ≤ 0.001. Scale bar: 20 μm.

Then, we perturbed the endogenous α-SNAP by microinjecting the negative dominant α-SNAP mutant, α-SNAP L294A. This mutant binds NSF but exhibits decreased ability to stimulate NSF's ATPase activity. α-SNAP L294A was produced in bacteria as a His6-tagged protein. When MII oocytes were microinjected with α-SNAP L294A previous to strontium activation, CGE was abolished (Fig 5A). This result strongly suggested that NSF´s ATPase activity is necessary for CGE. Interestingly, when wild type α-SNAP (also produced as a His6- recombinant protein) was microinjected, a partial inhibition of CGE was also observed. Even though it is accepted that α-SNAP disassembles the SNARE complex to regenerate SNARE members and make them available for membrane fusion, it has been documented that α-SNAP also binds syntaxin/SNAP-25 complex and free syntaxin [27–29]. Based on these observations and a recent report that showed that α-SNAP inhibits SNARE-mediated fusion of chromaffin granules in vitro [30], we hypothesized that the partial inhibition of CGE of wild type α-SNAP is due to its binding to free syntaxin (and/or syntaxin/SNAP-25 complex), interfering with the secretory process.

**Fig 6:**
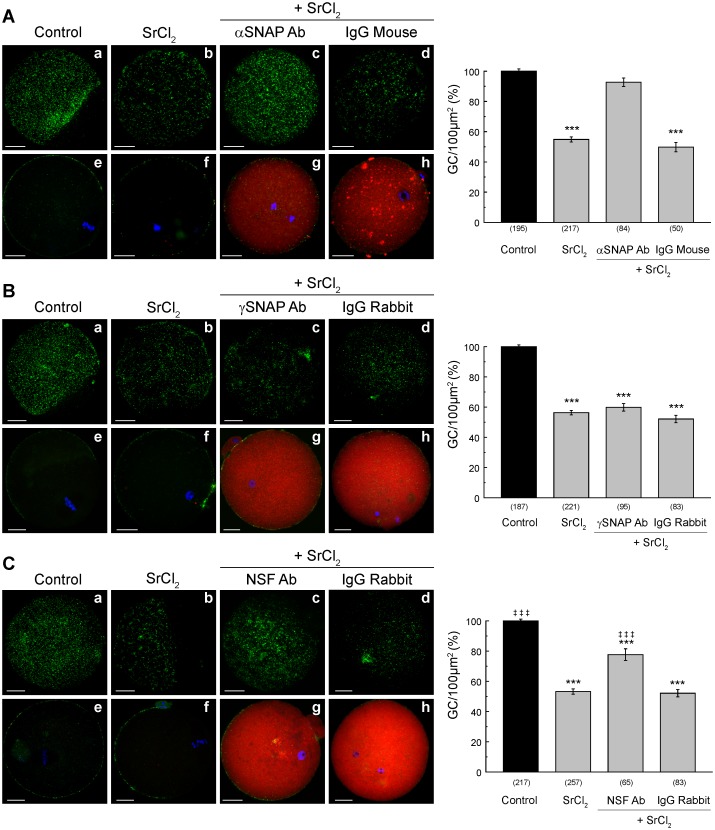
Effect of microinjection of α-SNAP, γ-SNAP and NSF antibodies on CGE. MII oocytes were microinjected with α-SNAP (**A**), γ-SNAP (**B**), and NSF (**C**) antibody and CGE was triggered with 30mM strontium chloride (SrCl_2_). Mouse and rabbit IgGs were microinjected as isotype controls at the same concentrations. *Left*: **a**-**d**, representative images of cells stained with FITC-LCA to label CG. **e**-**h**, representative images of oocytes subjected to inmunofluorescence protocol, were primary antibody was omitted, and secondary antibodies: Cy3 donkey anti-mouse in **A**, and Cy3 donkey anti-rabbit in **B** and **C**, were used to confirm proper microinjection of antibody or control isotype (red). *Right*,Histogram showing % CG density/100 μm^2^ relative to untreated group (Control) set as 100%. Data are shown as mean ± SEM from at least 4 independent experiments; numbers in parentheses below bars represent total number of oocytes. ***, values compared to control, p ≤ 0.001;‡ ‡ ‡, values compared to SrCl_2_, p ≤ 0.001. Scale bar: 20 μm.

Todemonstrate the participation of α-SNAP in cortical reaction, we microinjected the same antibody used in the previous assays of western blot and immunofluorescence. Mouse MII oocytes were microinjected with anti- α-SNAP prior strontium activation. Then, zona pellucida of the treated oocytes was removed before fixation and cortical granules were stained with FITC-Lens Culinaris Agglutinin (LCA) to evaluate cortical granule density. As shown in Fig 6A, the microinjection of anti-α-SNAP inhibited CGE. The microinjection of a mouse IgG isotype control had no effect, showing that microinjection procedure or an unspecific IgG were not responsible of the observed inhibition. Hence, these results indicate that α-SNAP has an active role and participates in cortical reaction.

Then, to test the hypothesis that γ-SNAP participates in cortical reaction, we performed similar experiments and microinjected the same anti-γ-SNAP antibody used previously to inhibit endogenous γ-SNAP during the activation of CGE. In this case, a rabbit IgG was microinjected as a control. The microinjection of anti-γ-SNAP antibody in MII oocytes prior parthenogenetic activation was not able to inhibit CGE activated by strontium chloride (Fig 6B), indicating that γ-SNAP does not have a role in this secretory process. To the extent of our knowledge the function of γ-SNAP has been poorly explored. There is an evidence that γ-SNAP, like α-SNAP, stimulates calcium-dependent exocytosis in adrenal chromaffin cells [25], but, unlike α-SNAP, γ-SNAP can bind NSF independently of SNAREs [26]. It is known that γ-SNAP specifically interacts with γ-SNAP associated factor-1 (Gaf-1) [31] and that overexpressed GFP-γ-SNAP colocalizes with Gaf-1 in mitochondria and microtubules in HEK-293 cells [31]. Hence, the roles of γ-SNAP in mouse oocytes remains to be explored.

To test the role of NSF in CGE, we first inhibited NSF function with N-ethylmaleimide (NEM), an alkylating reagent that irreversibly inhibits the ATPase activity of NSF. As shown in Fig 5B, pretreatment of MII oocytes with NEM abolished the strontium-induced CGE in a concentration-dependent manner. It is worthwhile to mention that the maximum inhibitory concentration assayed in oocytes (50 μM) was twenty times lower than NEM concentration used in the analysis of NSF role in acrosomal exocytosis in human sperm (1mM) [32] and neuronal secretion [5]. Because NEM is not specific for NSF and other NEM-sensitive factorshave been implicated in membrane fusion, it was importantto specifically perturb endogenous NSF protein. For this, we purified the negative mutant NSF D1EQ, in which glutamic acid 320 has been changed to glutamine residues in the D1 domain [19]. This NSF mutant is defective in ATP hydrolysis and cause a 70–80% decrease in ATPase activity related to wild type NSF. The microinjection of NSF D1EQ in mouse MII oocytes prior strontium activation inhibited significantly CGE (Fig 5C); nevertheless the microinjection ofwild type NSF had no effect on CGE assay (Fig 5C). The ATP hydrolysis mutant D1E-Q is able to bind to SNARE complex as does wild type NSF; however, it is not able to disassemble this complex. Our results showed that the ATPase activity of NSF is crucial for CGE.

Finally, to demonstrate that NSF is required for cortical granules secretion, we inhibited the function of the endogenous NSF by microinjecting the antibody raised against NSF. Fig 6C shows that anti-NSF antibody specifically inhibited cortical reaction stimulated by strontium, while the rabbit IgG isotype control had no effect in the cortical reaction. These results demonstrate that, like α-SNAP, NSF has an active role and is required for CGE.

### A working model for α-SNAP and NSF in cortical reaction of mouse oocytes

**Fig 7:**
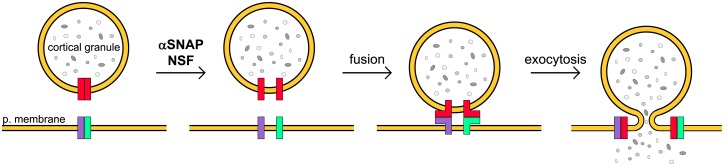
Working model for α-SNAP and NSF in CGE. When MII oocyte is activated by fertilization or by parthenogenesis, α-SNAP (α SNAP) and N-ethilmaleimide sensitive factor (NSF) disassemble cis-*SNARE* complexes (red, in cortical granules; green and purple, in plasma membrane) making their components available to allow the formation of trans-SNARE complex (red-purple and red-green complexes) for membrane fusion and secretion. p. membrane: plasma membrane.

Cortical granules only fuse once with plasma membrane after fertilization or parthenogenetic activation and are not renewed after this secretory process. There is no evidende about whether SNAREs are forming cis-SNARE complexes in mammalian oocytes [33]. Considering the two models proposed for α-SNAP and NSF function—the pre-fusion and the post-fusion model- during membrane fusion and, on the basis of the results presented here, we propose a working model for cortical granules exocytosis in mouse MII oocytes (Fig 7). Because we can inhibit CGE blocking α-SNAP and NSF function by mutants and antibodies microinjection, we proposed that α-SNAP and NSF would be participating in the initial phase of a round cycle, disassembling cis-SNAREs complexes and making their components available to allow the formation of trans-SNARE complexes for membrane fusion during cortical reaction (Fig 7). However, the kinetic of CGE is unknown and we cannot discard that fusion of cortical granules with plasma membrane occurs in waves. If this scenario were true, a function of α-SNAP and NSF after secretion of the first waves of fused cortical granules might be possible.

The knowledge about the role of α-SNAP and NSF in exocytosis comes from nondividing cells. It has been poorly explored whether or not cells in interphase share the same molecular mechanism of membrane fusion with cells in division. Based on published studies that investigated the effects of knockdown/knockout or overexpression of SNAREs, α-SNAP and NSF proteins on the normal function of cells as well as their dysfunction in cell cycle signaling, it has been proposed that the α-SNAP and NSF would be a targetfor novel anti-cancer therapeutics, among others pathologies, [34,35]. From this point of view, we speculate that α-SNAP and NSF would also be a target for contraception.

In conclusion, the results of our study reveal for the first time that α-SNAP, γ-SNAP, and NSF are expressed in mouse oocytes and that α-SNAP and NSF are required for CGE. Further investigation will be necessary to elucidate the function of γ-SNAP in the progression of meiosis. Both, the membrane fusion during meiosis and α-SNAP and NSF as new therapeutic targets are emerging themes.

## Supporting Information

S1 Fig Scheme of the experimental design. The graph represents hormonal stimulation (PMSG, hCG) of mice to obtain Germinal Vesicle-intact oocytes (GV oocytes) and Metaphase II oocytes (MII oocytes), and the different methods used in this study.
(TIF)

S2 Fig α-SNAP, γ-SNAP, and NSF localization during different stages. α-SNAP (left panel), γ-SNAP (middle panel) and NSF (right panel) were immunodetected at different stages: GV-intact oocytes (GV), MII oocytes(MII), parthenogenetic activated MII oocytes with 10mM strontium chloride during 1 or 7 h post activation (SrCl_2_ (1h) and (7h), respectively), and two pronucleus (2PN) embryos after in vitro fertilization. Green, positive staining for primary α-/β-SNAP, γ-SNAP or NSF antibody detected by secondary antibodies conjugated to DyLight 488; red, cortical granules stained with LCA-Rhodamine; blue, DNA labeled with Hoechst 3342. Differential interference contrast (DIC) images are shown on the right of each panel. The last row of all panels shows negative cell staining when the primary antibody was not used. Scale bar: 20 μm.
(TIF)
