# Supplementary figures and images for: Cortical Granule Exocytosis Is Mediated by Alpha-SNAP and N-Ethilmaleimide Sensitive Factor in Mouse Oocytes
Source: PLoS One. 2015 Aug 12;10(8):e0135679. doi: 10.1371/journal.pone.0135679 (PMC4534440; doi:10.1371/journal.pone.0135679)

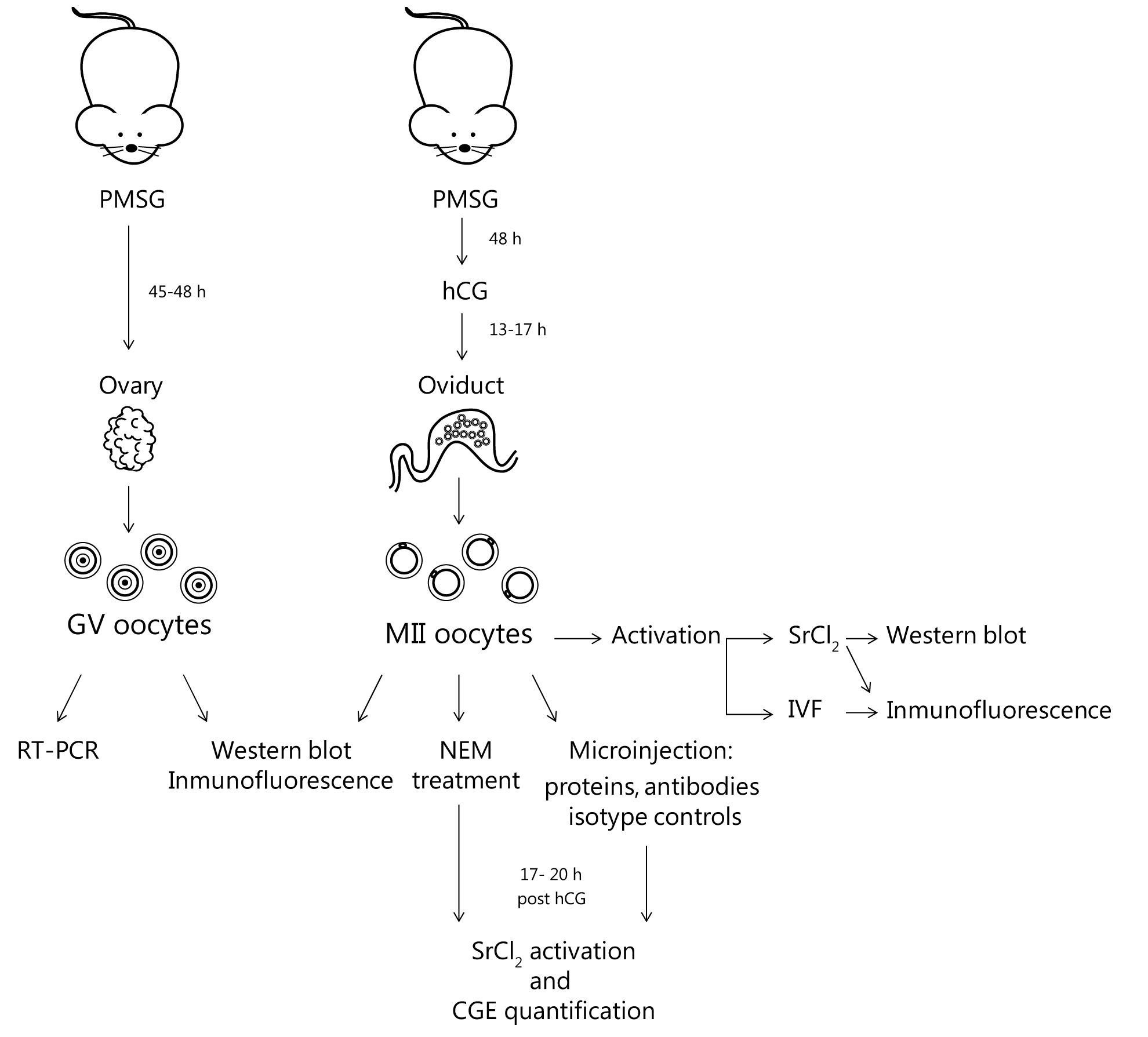

Supplement: S1 Fig — The graph represents hormonal stimulation (PMSG, hCG) of mice to obtain Germinal Vesicle-intact oocytes (GV oocytes) and Metaphase II oocytes (MII oocytes), and the different methods used in this study. (TIF) [file pone.0135679.s001.tif]

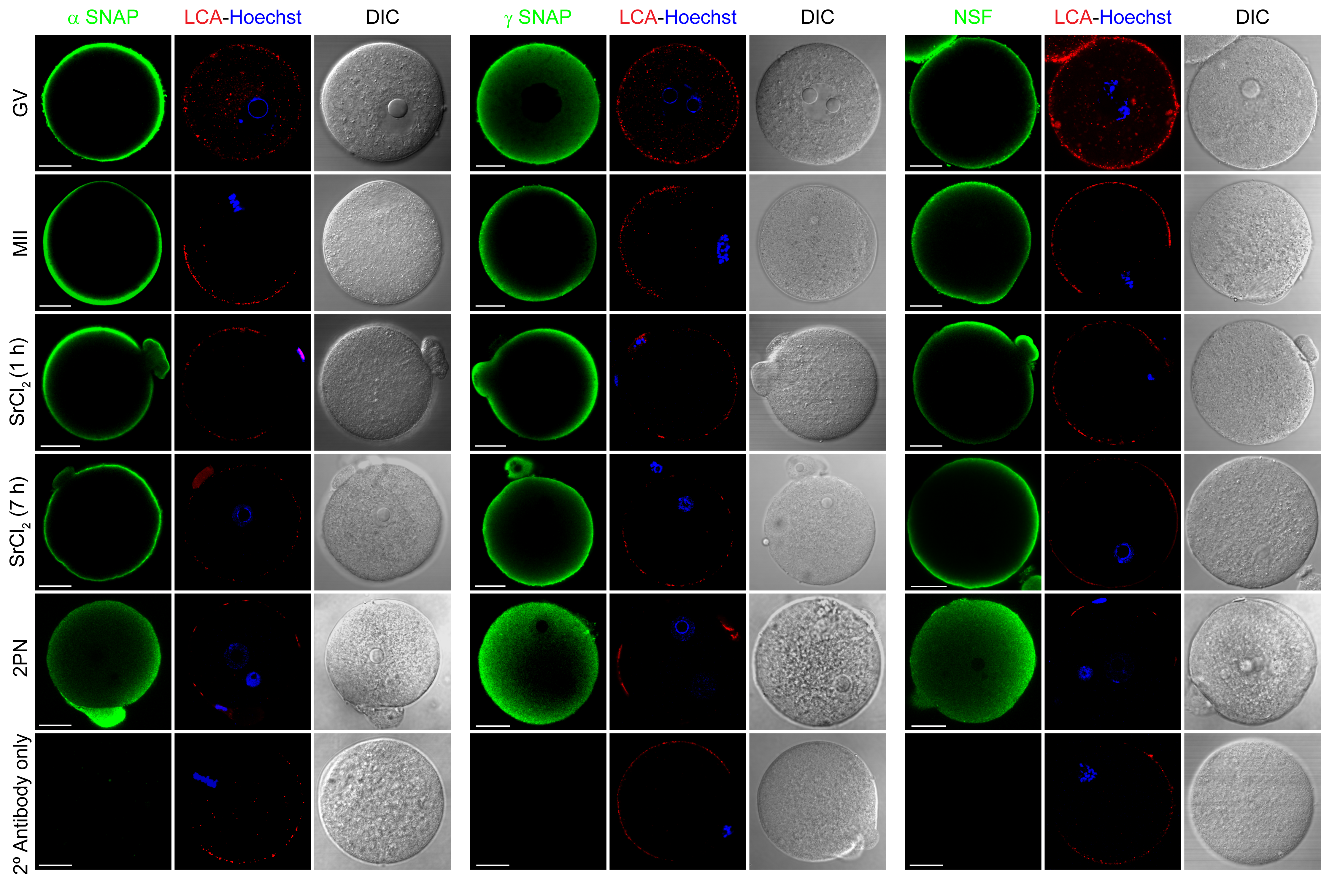

Supplement: S2 Fig — α-SNAP (left panel), γ-SNAP (middle panel) and NSF (right panel) were immunodetected at different stages: GV-intact oocytes (GV), MII oocytes(MII), parthenogenetic activated MII oocytes with 10mM strontium chloride during 1 or 7 h post activation (SrCl2 (1h) and (7h), respectively), and two pronucleus (2PN) embryos after in vitro fertilization. Green, positive staining for primary α-/β-SNAP, γ-SNAP or NSF antibody detected by secondary antibodies conjugated to DyLight 488; red, cortical granules stained with LCA-Rhodamine; blue, DNA labeled with Hoechst 3342. Differential interference contrast (DIC) images are shown on the right of each panel. The last row of all panels shows negative cell staining when the primary antibody was not used. Scale bar: 20 μm. (TIF) [file pone.0135679.s002.tif]
